# A Comparison of Vitamin E Status and Associated Pregnancy Outcomes in Maternal–Infant Dyads between a Nigerian and a United States Population

**DOI:** 10.3390/nu10091300

**Published:** 2018-09-14

**Authors:** Caleb Cave, Corrine Hanson, Marina Schumacher, Elizabeth Lyden, Jeremy Furtado, Stephen Obaro, Shirley Delair, Nicholas Kocmich, Amy Rezac, NI Izevbigie, Matthew Van Ormer, Ammar Kamil, Elizabeth McGinn, Katherine Rilett, Elizabeth Elliott, Rebecca Johnson, Kara Weishaar, EK Olateju, GA Akaba, EA Anigilaje, Tahiru Tahiru, Ann Anderson-Berry

**Affiliations:** 1 Pediatrics 981205 Nebraska Medical Center, University of Nebraska Medical Center, Omaha, NE 68198-1205, USA; caleb.cave@unmc.edu (C.C.); stephen.obaro@unmc.edu; (S.O.); shirley.delair@unmc.edu (S.D.); nick.kocmich@unmc.edu (N.K.); amy.rezac@unmc.edu (A.R.); sakharemog@yahoo.co.uk (N.I.); matthew.vanormer@unmc.edu (M.V.O.); dr.ammar.kamil@gmail.com (A.K.); elizabeth.mcginn@unmc.edu (E.M.); katherine.rilett@unmc.edu (K.R.); elizabeth.elliott@unmc.edu (E.E.); rebecca.johnson@unmc.edu (R.J.); kara.weishaar@unmc.edu (K.W.); 2 984045 Nebraska Medical Center, College of Allied Health Professions, Medical Nutrition Education, University of Nebraska Medical Center, Omaha, NE 68198-4045, USA; ckhanson@unmc.edu (C.H.); marinaschumacher@me.com (M.S.); 3 984375 Nebraska Medical Center, College of Public Health, University of Nebraska Medical Center, Omaha, NE 68198-4375, USA; elyden@unmc.edu; 4 Department of Nutrition, Harvard School of Public Health, 655 Huntington Avenue, Boston, MA 02215, USA; jfurtado@hsph.harvard.edu; 5 University of Abuja Teaching Hospital, Gwagwalada-Zuba, Gwagwalada, P.M.B. 228, Abuja, Nigeria; oeyinade@yahoo.com (E.O.); docakabago@yahoo.com (G.A.); naijanannytales@yahoo.com (E.A.); tahirosma@yahoo.com (T.T.)

**Keywords:** vitamin E, tocopherols, maternal, infant, pregnancy, Nigeria, United States

## Abstract

Oxidative stress is associated with adverse pregnancy outcomes, and vitamin E has powerful anti-oxidant properties with the potential to impact health outcomes. Tocopherol isomers of vitamin E differ in their ability to modulate inflammation and vary in concentration in diets containing high proportions of processed versus unprocessed foods. The purpose of this study was to compare vitamin E status and associated pregnancy outcomes (mode of delivery, chorioamnionitis, APGARs (measure of appearance, pulse, grimace, activity, respiration), gestational age at delivery, and fetal growth) between maternal–infant dyads in a developed and a developing nation to identify potentially modifiable differences that may impact pregnancy and neonatal outcomes and provide a way to improve maternal and neonatal health. Plasma tocopherol levels were evaluated in 189 Midwestern United States (US) mother–infant pairs and 99 Central Nigerian mother–infant pairs. Maternal and infant concentrations of α-, γ-, and δ-tocopherol were measured using HPLC with diode-array detection. Descriptive statistics were calculated and tocopherol concentrations were associated with clinical outcomes such as mode of delivery, chorioamnionitis, APGARS, and fetal growth. Alpha- and γ-tocopherol levels were higher in the US mothers, (alpha: 12,357.9 (175.23–34,687.75) vs. 8333.1 (1576.59–16,248.40) (mcg/L); *p* < 0.001) (gamma: 340.7 (224.59–4385.95) vs. 357.5 (66.36–1775.31) (mcg/L); *p* < 0.001), while δ-tocopherol levels were higher in the Nigerian mothers (delta: 261.7 (24.70–1324.71) vs. 368.9 (43.06–1886.47) (mcg/L); *p* < 0.001). US infants had higher γ-tocopherol levels than Nigerian infants (203.1 (42.53–1953.23) vs. 113.8 (0.00–823.00) (mcg/L); *p* < 0.001), while both the Nigerian mothers and infants had higher α:γ-tocopherol ratios (8.5 vs. 26.2, and 8.9 vs. 18.8, respectively; *p* < 0.001). Our results in both populations show associations between increased circulating γ-tocopherol and negative outcomes like Caesarian sections, in contrast to the associations with positive outcomes such as vaginal delivery seen with increased α:γ-tocopherol ratios. Growth was positively associated with α- and γ-tocopherols in cord blood in the US population, and with cord blood δ-tocopherols in the Nigerian population. Tocopherol levels likely impact health outcomes in pregnancy in a complicated metabolism across the maternal–fetal axis that appears to be potentially influenced by culture and available diet.

## 1. Introduction

Vitamin E plays a protective role for both the mother and infant during pregnancy because of its role as a chain-breaking antioxidant and as the primary lipid peroxyl radical scavenger in the human body [1]. This protection is necessary since unbalanced oxidant stress during pregnancy may cause preeclampsia, preterm delivery, and low birth weight [2,3,4]. Sufficient levels of vitamin E in early infancy can also help prevent major complications in infants, such as bronchopulmonary dysplasia, intraventricular hemorrhage, and developmental delays of the central nervous system [5].

Vitamin E naturally occurs in several different tocopherol isomers (α, β, δ, and γ) which can also undergo acetylation [6]. Although γ-tocopherol is the most common form of vitamin E found in the Western diet, α-tocopherol has higher levels in human tissues, due to increased degradation and excretion of γ-tocopherol, and increased deposition of α-tocopherol into cell membranes and lipoprotein–lipid bundles by α-tocopherol transfer protein (α-TTP) [6,7,8]. At equivalent concentrations, both α-tocopherol and γ-tocopherol possess comparative antioxidant abilities as scavengers of reactive oxygen species [9,10]. However, γ-tocopherol also has pro-inflammatory properties in the human body since it reacts with reactive nitrogen species, such as those that help drive neutrophilic inflammation [11,12,13]. These pro-inflammatory properties of γ-tocopherol are strong enough to negate the anti-inflammatory properties of α-tocopherol, even when plasma concentrations of γ-tocopherol are as low as 10% of α-tocopherol plasma levels [14]. This makes evaluation of α:γ-tocopherol ratios, in addition to analysis of individual tocopherols, important in understanding the impact on clinical conditions. Little is currently known about the β and δ isoforms.

Overt vitamin E deficiency is incredibly rare in the United States (US) [1]. However, according to the Food and Agriculture Organization (FAO) of the United Nations, no information (as of 2011) exists on vitamin E deficiency in developing countries on the African continent [15]. Also, to our knowledge, little work was done comparing vitamin E status and plasma tocopherol levels between populations of a developed country and a developing country to expose geographical nutritional disparities that may stem from cultural and economic factors. As tocopherol levels were demonstrated to impact important pregnancy outcomes, and levels are directly impacted by diets high in processed foods, as those often found in the US, but less prevalent in developing countries, evaluation of tocopherol status in pregnant women and their infants in these two different cultures has the potential to inform development of dietary intervention to help optimize diets in pregnancy and positively impact maternal and infant outcomes. Evidence that tocopherol levels, particularly α-tocopherol, are associated with pregnancy outcomes, such as fetal growth, is well documented [16,17,18,19]. Therefore, comparing plasma tocopherol levels with selected pregnancy outcomes such as length of gestation, development of preeclampsia, fetal growth, pregnancy loss, and other maternal and fetal factors between United States and Nigerian maternal–infant populations could illuminate the need for nutritional interventions to positively impact adverse pregnancy outcomes in high-risk populations.

The purpose of this study was to compare vitamin E status and associated pregnancy outcomes (mode of delivery, chorioamnionitis, APGARs (measure of appearance, pulse, grimace, activity, respiration), and fetal growth) between maternal–infant dyads in a developed and a developing nation to identify potentially modifiable differences that may impact pregnancy and neonatal outcomes and provide a way of improving maternal and neonatal health.

## 2. Materials and Methods

### 2.1. Recruitment

This was a comparison of two cross-sectional populations evaluating vitamin E status of 189 United States maternal–infant pairs and 99 Nigerian maternal–infant pairs. US subjects were recruited from the Nebraska Medicine Labor and Delivery unit, Newborn Nursery, and the Newborn Intensive Care unit in Omaha, Nebraska, United States, an academic medical center serving a delivery population with racial demographics that closely mirror the racial demography of the United States population. Nigerian subjects were enrolled while attending the antenatal clinic or delivery center at the University of Abuja Teaching Hospital in Gwagwalada, Nigeria.

### 2.2. Ethical Approvals

Separate institutional review board (IRB) approvals were obtained from the University of Nebraska Medical Center for participant enrolment in the United States and Nigeria as the study protocols were different due to health system and cultural differences. In addition, ethical approval for the Nigerian cohort was granted from the University of Abuja Teaching Hospital, Gwagwalada in Abuja, Nigeria.

### 2.3. Sample and Data Collection

Samples of both cord and maternal blood were collected at the time of delivery from those who consented to participate. Relevant clinical data were collected from both populations. Inclusion criteria for the US population were all live births at the Nebraska Medicine Labor and Delivery Unit to mothers aged 19 or older and able to give informed consent in English or with a medical interpreter. Exclusion criteria for the US population included congenital abnormalities, gastrointestinal (GI), liver, or kidney disease, or inborn errors of metabolism in the infant or the mother. Inclusion criteria for the Nigerian population included maternal age ≥18 years, gestational age ≥24 weeks, infant age 0–7 days at enrolment (however, all mother–infant dyads were enrolled at or before delivery), absence of heavy peripartal vaginal bleeding, and ability to provide written informed consent. No other specific exclusion criteria were stated for the Nigeria cohort. Pertinent demographic and clinical data including but not limited to maternal age, weight, body mass index (BMI), smoking status, gravidity and parity, concomitant maternal diagnoses, mode of delivery, medications, food frequency questionnaire (FFQ), and infant birth anthropometrics, APGARs, feeding status, and neonatal intensive care unit (NICU) medical history if applicable, were collected from the US population’s electronic medical records. Demographic and clinical data from the Nigerian subjects were prospectively collected and entered into the Midwestern academic medical center electronic capture tools, collectively termed REDCap. As part of this data collection, a 28-day follow-up phone interview was conducted with participating Nigerian mothers to assess infant well-being and interval illness or hospitalization. Clinical data collected from both populations included maternal age, BMI, smoking status, chorioamnionitis diagnoses, infant corrected gestational age, birth anthropometrics, gender, and APGAR scores (1-min and 5-min).

### 2.4. Nutrient Intake Data

The US recommended dietary allowance of vitamin E for pregnant women is 15 mg/day of α-tocopherol [1]. There is not a validated tool for the Nigerian diet; therefore, no intake information is available for the cohort and intake is not addressed in this analysis. Maternal intake data were collected in the US population by administering the Willett food frequency questionnaire (FFQ) to all maternal participants at the time of delivery. An FFQ was used to assess vitamin E intake to best estimate intake over time. This is currently the most reliable validated nutrition tool to assess nutrient intake during the course of a pregnancy. An accurate representation of vitamin E intake over the course of several days is preferred over a 24-h diet recall [20,21]. FFQs were analyzed at the Harvard School of Public Health (Boston, MA, USA) [22].

### 2.5. Biochemical Analysis

Blood samples were collected in ethylenediaminetetraacetic acid (EDTA) tubes at the time of the mother’s admission in labor, and from the cord at the time of delivery. All samples were protected from heat and light, centrifuged and aliquoted, and frozen at −80 °C within a maximum of 12 h. Measurements of plasma concentrations of α-tocopherol, γ-tocopherol, and δ-tocopherol were obtained using high-performance liquid chromatography with diode-array detection. Concentrations of α-, γ-, and δ-tocopherol in plasma samples were measured as described by El-Sohemy et al., who found plasma levels to correlate well with fat tissue samples using this method [23]. Plasma samples were mixed with ethanol containing 10 mg/mL rac-tocopherol (Tocol) as an internal standard, extracted with hexane, evaporated to dryness under nitrogen, and reconstituted in ethanol–dioxane (1:1 *v*/*v*) and acetonitrile. Samples were quantitated by high-performance liquid chromatography (HPLC) on a Restek Ultra C18 150 mm × 4.6 mm column, 3-mm particle size encased in a column oven (Hitachi L-2350, Hitachi, San Jose, CA, USA) to prevent temperature fluctuations, and equipped with a trident guard cartridge system (Restek, Corp. Bellefonte, PA, USA). A mixture of acetonitrile, tetrahydrofuran, methanol, and a 1% ammonium acetate solution (68:22:7:3) was used as mobile phase at a flow rate of 1.1 mL/min, with a Hitachi L-2130 pump in isocratic mode and a Hitachi L-2200 auto-sampler with water-chilled tray. Detection of the analytes was achieved using a Hitachi L-2455 diode-array detector monitoring ultraviolet and visible (UV/Vis) wavelengths: 300 nm for retinol and the tocopherols, and 445 nm for the xanthophyls, lycopene, and carotenoids. The Hitachi System Manager software (D-2000 Elite, Version 3.0) was used for peak integration and data acquisition. Calibration curves were created for each analyte using commercially available pure standards (Millipore Sigma, Burlington, MA, USA; Matreya LLC, State College, PA, USA; Indofine Chemical, Hillsborough, NJ, USA; DHI USA, Lakewood, CO, USA) dissolved into known-concentration working solutions that were quantified by UV/Vis analysis using spectral coefficients obtained from the Merck Index and CRC Handbook of Chemistry and Physics, which were then serially diluted to cover physiological ranges. These curves were then used to analyze standard reference material from the National Institute of Standards and Technology (NIST; SRM 968), which provides certified and reference values for all of the analytes in our panel. Obtained values must fall within the range if the NIST values.

Internal quality control was monitored with four large-volume plasma pool control samples analyzed within each run. These samples were collected from volunteers at the Harvard School of Public Health whose plasma was anonymized and assigned to either a high-level pool or a low-level pool, both of which were then calibrated against NIST SRM 968. Two samples of the high-level pool and two samples of the low-level pool were run in each batch to allow calculation of within-run and between-run variation estimates. In addition, external quality control was monitored by participation in the standardization program for carotenoid analysis from the National Institute of Standards and Technology USA. Using this method, β-tocopherols cannot be detected, and therefore, were not evaluated in this study.

### 2.6. Growth Analysis

Birth growth parameter percentiles were constructed for infant birth weight, length, and head circumference using international standards from the INTERGROWTH-21st Project [24]. Birth growth parameter z-scores were constructed using the World Health Organization (WHO) Child Growth Parameter’s Anthro software for SPSS (SPSS Inc., Chicago, IL, USA).

### 2.7. Statistical Analysis

Data were summarized using means, standard deviations, medians, ranges, counts, and percentages. Spearman correlation coefficients were used to look at the association of maternal and cord blood measurements, as well as correlation of plasma tocopherol levels with select clinical outcomes. Mann–Whitney tests were used to compare median plasma values between dichotomous variables. Statistical significance was set at *p* ≤ 0.05. SAS version 9.3 (SAS Institute, Cary, NC, USA) was used for all analysis.

## 3. Results

### 3.1. Baseline Characteristics

In the two studies, 189 United States maternal–infant pairs and 99 Nigerian mother-infant pairs were included as subjects. Of the US subjects, 180 mothers and 173 infants had plasma samples analyzed at the time of delivery. Ninety-nine Nigerian mother-and-infant plasma sample pairs from the time of delivery were analyzed. Prematurity was defined as a corrected gestational age ≤37 weeks (Table 1).

### 3.2. Maternal and Infant Population Plasma Levels

The mean δ-tocopherol plasma levels were higher in the Nigerian maternal population than in the Midwest maternal population. However, the mean α-tocopherol and γ-tocopherol levels were lower in the Nigerian mothers than in the corresponding US maternal population. Vitamin E deficiency was defined as plasma α-tocopherol levels <5186 mcg/L (516 mcg/dL), and vitamin E status for the United States population was replete based on reference values from the Institute of Medicine where significant hemolysis was noted in adult men with levels lower than this threshold. Gender- and age-specific cut-offs are not available [1]. In contrast, using the same reference values, vitamin E deficiency affected 9.2% of Nigerian mothers and 91.89% of Nigerian infants. The maternal Nigerian population α:γ-tocopherol ratio was significantly higher than the α:γ-tocopherol ratio of the United States maternal population.

The Nigerian infant population had higher plasma α-tocopherol and δ-tocopherol levels than the US infant population, while the US infant population had higher γ-tocopherol levels. These values subsequently contributed to a higher α:γ-tocopherol ratio in the Nigerian infant population over the Midwestern population (Table 2).

### 3.3. Tocopherol Correlations with Maternal and Infant Characteristics

Cord levels of γ-tocopherol from the United States infant population were positively correlated with maternal pre-pregnancy BMI (R = 0.29, *p* = 0.003). None of the cord or maternal plasma tocopherol levels correlated with maternal pre-pregnancy BMI in the Nigerian population. Although maternal intake of α-tocopherol in the US maternal population demonstrated a trend toward a positive association with cord α-tocopherol concentrations (R = 0.17, *p* = 0.06), maternal intake of α-tocopherol in the US was inversely associated with maternal plasma concentrations of both γ-tocopherol and δ-tocopherol (R = −0.20, *p* = 0.02, and R = −0.27, *p* = 0.002, respectively).

The US infant population cord levels of γ-tocopherol were inversely associated with corrected gestational age (R = −0.17. *p* = 0.03), while none of the population’s other maternal or cord tocopherol levels had any correlations with corrected gestational age. None of the Nigerian cord or maternal plasma tocopherol levels were associated with corrected gestational age. Infant gender was not significantly associated with any maternal or cord tocopherol levels in either the US population or the Nigerian population.

### 3.4. Maternal–Cord Transfer of Vitamin E Tocopherols

There was no significant correlation between maternal and infant α-tocopherol levels in either population. Gamma-tocopherol maternal and infant levels were positively correlated in both populations. Delta-tocopherol maternal and cord-blood plasma concentrations were correlated in the US population, but not in the Nigerian population.

### 3.5. Associations with Maternal Clinical Outcomes

Clinical outcomes of maternal subjects in each population were defined as mode of delivery (Caesarean section vs. vaginal delivery) and the presence or absence of a diagnosis of chorioamnionitis. In the US maternal population, concentrations of γ-tocopherol were higher in mothers who delivered via Caesarean section compared to vaginal delivery. Within the Nigerian population, maternal levels of γ-tocopherol and cord-blood plasma γ-tocopherol, as well as δ-tocopherol, concentrations were both significantly higher in infants delivered by Caesarean section than by vaginal deliveries (Table 3).

US maternal levels of α-tocopherol and γ-tocopherol were higher in mothers without a diagnosis of chorioamnionitis compared to those diagnosed with chorioamnionitis (showing a trend toward significance, *p* = 0.058 and *p* = 0.051, respectively; Table 4). Since there were no recorded diagnoses of chorioamnionitis in the Nigerian population (82% were negative for chorioamnionitis, while the remaining 18% had an unknown diagnosis), correlations between maternal and infant tocopherol levels with this clinical outcome could not be calculated.

### 3.6. Associations with Infant Clinical Outcomes

Clinical outcomes of infant subjects were defined as gestational age at delivery, low APGAR scores (<7) at 1 min and 5 min (APGARs are universally assigned and are a standardized way of describing an infant’s stability as they transition from the intrauterine to extra-uterine environment by scoring heart rate, respiratory effort, tone, and oxygenation, making this a good marker to use between these populations), as well as the birth growth parameters of length, weight, and head circumference (dichotomized at the 10th percentile for each parameter), and weight-for-length, weight-for-age, length-for-age, and head-circumference-for-age (dichotomized at a z-score of −2). Z-scores were calculated using the WHO infant growth parameter standards. Z-scores for weight-for-length and weight-for-age were calculated for both populations, with length-for-age being calculated for the United States population only and head-circumference-for-age for the Nigerian population only.

Within the United States population, no maternal or cord tocopherols correlated with gestational age at delivery. Maternal plasma levels of α-tocopherol were positively associated with 5-min APGAR scores (R = 0.16936, *p* = 0.026), while cord levels of α-tocopherol demonstrated an inverse relationship with 5-min APGAR scores (R = −0.16542, *p* = 0.033). None of the maternal or cord plasma tocopherol levels within the Nigerian population were significantly associated with APGAR scores, although there was a statistically significant inverse relationship between 5-min APGAR scores and cord α:γ-tocopherol ratio (R = −0.27154, *p* = 0.024).

In the US population, higher maternal α-tocopherol levels were associated with birth length >10th percentile, while higher cord levels of α-tocopherol were associated with birth length <10th percentile (Table 5). Using Z-scores >−2 standard deviations below the mean to evaluate growth between the two populations revealed significant associations between cord tocopherols, as described in Table 5.

## 4. Discussion

### 4.1. Baseline Characteristics

The major difference noted in the two cohorts were likely based on differences in cultural norms and healthcare implementation between the two countries. Smoking during pregnancy remains much higher than ideal in the US, and it is apparent that the diagnosis of chorioamnionitis is an infrequent one in the Nigerian hospital where the study was performed. Moreover, an evaluation of the differences in diets between the two countries would be helpful. To date, a validated tool to assess nutrient intake such as the FFQ used in the US is not available for Nigeria. Differences in diet composition in the Nigerian maternal and infant populations when compared to the United States populations may have a foundation in a complex combination of culture, religion, climate, and socioeconomic differences. A concern in the US diet is the proliferation of processed foods high in γ-tocopherol and lower in α-tocopherol. In developing countries such as Nigeria, there is likely to be less processed food intake; however, as the country industrializes, this can change rapidly. A more comprehensive understanding through careful evaluation of α- and γ-tocopherol concentrations, ratios, and compound transition across the maternal–fetal axis is critical in order to better understand the impact of diet on maternal fetal health. Understanding the differing cellular actions these compounds may have and their impact on inflammation will be critical in elucidating what recommendations may be necessary for diet during and while preparing for pregnancy.

### 4.2. Maternal and Infant Plasma Levels

The decreased γ-tocopherol levels in the Nigerian mothers and infants contributed to an overall higher α:γ-tocopherol ratio in the Nigerian population, which led to a more favorable vitamin E anti-inflammatory profile. The differences in maternal tocopherol levels between the two populations are best explained by suspected dietary differences based on knowledge of cultural differences and the US FFQ data. The typical Nigerian diet is more plant-based (green, leafy vegetables and nuts are higher in α-tocopherol), while the typical US diet includes many foods cooked with or containing canola, soybean, and corn oils (foods higher in γ-tocopherol) [25,26]. Additionally, foods high in vitamin C and β-carotene, such as those found in the Nigerian diet, were also noted to have synergistic mechanisms of action with α-tocopherol, recycling α-tocopherol back to its more potent antioxidant form [27]. Lower overall α-tocopherol levels in the Nigerian mothers could be due to influences of food insecurity and living in a high-risk socioeconomic environment as compared to the mothers in the US cohort.

### 4.3. Tocopherol Correlations with Maternal and Infant Characteristics

BMI and cord γ-tocopherol were positively associated, while the same value was inversely associated with corrected gestational age, indicating an association with poor health, increasing fat mass and this fat-soluble molecule. With nutrition studies, all associations need to be evaluated in light of the significant confounders in other nutrients, medications, lifestyle, and other possibly unknown factors. It can be impossible to adjust for these confounders in small preliminary studies such as this; however, to note the associations for further evaluation in subsequent investigations is critical.

### 4.4. Maternal–Cord Transfer of Vitamin E Tocopherols

Maternal–fetal transfer of γ-tocopherol was suggested by the correlation between maternal and infant γ-tocopherol levels in both populations. This correlation is supported by a previous study performed by Yeum et al. in 1998 [28]. We question if the high-γ-tocopherol diet in the US allows this molecule to pass freely through the placenta while α- and δ-tocopherol compounds may be regulated or utilized by the placenta to modulate pregnancy-associated inflammation for fetal protection. This would explain why the lower levels of δ-tocopherol as seen in the Nigerian population do not demonstrate correlation between maternal and cord samples, while we do see some correlation in the US samples where the levels are higher. There was no significant correlation between maternal and infant α-tocopherol levels in either population to suggest significant direct maternal–fetal α-tocopherol transfer. These results are supported by a prior study performed by Keily et al. evaluating plasma levels of α-tocopherol and γ-tocopherol concentrations in 40 mother–infant pairs, finding a correlation between γ-tocopherol levels in the mothers and infants (R = 0.45, *p* = 0.0005), but no association between concentrations of α-tocopherol [29]. One explanation for the lack of placental transfer of α-tocopherol is its uniquely high affinity, among the tocopherols, for α-TTP [30]. If the α-TTP protein system is underdeveloped in infants, it could lower circulating levels of α-tocopherol in the cord blood [31]. Another possible explanation for low α-tocopherol in infants is that α-tocopherol is deposited into tissues following birth, reducing its plasma levels. This idea is supported by a study performed in a lamb model, in which lambs born to α-tocopherol-supplemented ewes displayed low plasma α-tocopherol levels, but elevated α-tocopherol levels in brain and muscle tissue [32]. Evaluation of placental tissue levels of tocopherols may be helpful in further understanding this process.

### 4.5. Associations with Maternal Clinical Outcomes

Mode of delivery is an important pregnancy outcome, as vaginal delivery is associated with decreased maternal and infant mortality and morbidity, as well as decreased length of stay in the US. Concentrations of γ-tocopherol in both US and Nigerian maternal populations were higher in mothers who delivered via Caesarean section compared to vaginal delivery, posing an interesting association between a potentially pro-inflammatory molecule and a decreased ability to have a natural delivery. While Nigerian cord-blood γ-tocopherol and δ-tocopherol concentrations were significantly higher in subjects delivering by Caesarean section than by vaginal delivery, no association with plasma maternal α-tocopherol levels with mode of delivery was previously illustrated in a Brazilian maternal population. In this study, levels of α-tocopherol in maternal plasma and colostrum samples were positively associated with elective Caesarean delivery [33]. Higher levels of γ-tocopherol in mothers delivering by Caesarean section may be related to both diet-mediated inflammation and the underlying pro-inflammatory conditions in those mothers, leading to a delivery by Caesarean section [31].

The diagnosis of chorioamnionitis is common in the US; however, a lack of recorded diagnoses of chorioamnionitis in the Nigerian population prevented evaluating associations and population differences with tocopherol levels. It is worth noting, however, that α- and γ-tocopherol concentrations in the US maternal population were higher in US mothers without chorioamnionitis, an inflammatory condition caused by infection of the fetal membranes during pregnancy. It is possible that the decrease in maternal α- and γ-tocopherol levels in those with chorioamnionitis is compensatory, i.e., γ-tocopherol levels decrease as they are used to help drive acute neutrophilic inflammation in response to a bacterial infection, and α-tocopherol levels decrease as they counteract or are ablated by the harmful oxidant stress of the acute inflammation. Also, low baseline levels of anti-inflammatory α-tocopherols could increase maternal susceptibility to infection and inflammation including chorioamnionitis. Further studies should be done to elicit the role of tocopherols in chorioamnionitis and fetal infection, as this has a big impact on infant mortality, particularly in developing countries.

United States maternal α-tocopherol levels were directly correlated with 5-min APGAR scores, while US cord-blood α-tocopherol levels were inversely correlated with 5-min APGAR scores. The Nigerian population did not mirror these results, yet Nigerian infant plasma α:γ-tocopherol ratios were inversely correlated with 5-min APGAR scores. Maternal correlation of α-tocopherol levels with APGAR scores is supported by a randomized study of placebo vs. vitamin E supplement, in which pregnant women given vitamin E showed α-tocopherol levels trending toward an association with 5-min APGAR scores (*p* = 0.09) [34]. The inverse relationship of infant α-tocopherol concentrations with APGAR scores could represent decreased tissue uptake of α-tocopherol by the fetus [31]. Further investigation into the placental transfer and tissue uptake of cord α-tocopherol is warranted to better explain its relationship with infant outcomes.

### 4.6. Associations with Infant Clinical Outcome

Fetal and infant growth is routinely used as a surrogate for pregnancy and infant health, and poor growth is known to correlate with increased mortality and morbidity and poorer neurodevelopmental outcomes. The United States cord α- and γ-tocopherol levels and Nigerian cord δ-tocopherol levels were all associated with better infant growth in at least one parameter (weight, length, and head circumference, respectively), suggesting that vitamin E modulated growth either directly at a cellular level or by decreasing inflammation in utero allowing for more optimal growth.

### 4.7. Limitations

Limitations of this study include a relatively small sample size in both populations and an inability to quantify nutrient intake in the Nigeria population to compare to the FFQ. While validated for a pregnant population, the FFQ is not perfect in giving precise intake; however, it is the best tool to estimate intake over a longer time frame like a nine-month gestation. Healthcare and cultural differences, including the lack of diagnoses of chorioamnionitis in the Nigerian population, and an almost non-existent smoking population among the Nigerian subjects, make more complete analysis difficult. Smoking was shown to increase oxidant stress and is a known confounding factor when measuring serum vitamin E levels [35].

## 5. Conclusions

While the Nigerian population displayed a higher incidence of vitamin E deficiency than their United States counterparts, they also had higher α:γ-tocopherol ratios leading to an overall positive vitamin E inflammatory profile. This is important information given both the US and Nigeria study population rates of prematurity and Caesarian section delivery were similar despite system differences in healthcare resources and disparate national economic status in a developed vs. a developing country. A higher proportion of anti-inflammatory activity as shown by the α:γ-tocopherol ratio could easily contribute to the better than predicted outcomes of the Nigerian mothers and infants. Appropriate interventions for the Nigerian population could involve standardized provision of unprocessed foods high in α-tocopherol as opposed to vitamin E supplementation for pregnant maternal populations. Given the known low economic status of most pregnant women in Nigeria seeking care in a public hospital, supplementation directly with food should be successful, although there is a possibility it could be sold or given to the father or more senior wives for consumption. This diet augmentation may have the added bonus of reducing levels of γ- and δ-tocopherol levels [36]. As for the US maternal population, dietary interventions should include recommendations to increase consumption of raw foods high in α-tocopherol and decrease consumption of foods prepared with or containing oils high in γ-tocopherol, although we acknowledge that diet modification in any population is very challenging. Clinical outcomes point toward the benefits of increased levels of maternal α-tocopherol and decreased levels of maternal γ-tocopherol. Further research into the role of δ-tocopherol in humans and the placental transport and uptake of all tocopherols into developing fetuses could assist in better understanding the impact on pregnancy outcomes of dietary tocopherols and mediation through inflammatory modulation.

## Figures and Tables

**Table 1:** Maternal and infant demographics.

	**United States Maternal Population**		**Nigerian Maternal Population**		***p*-Value**
*Continuous variables:*	*N*	*Mean (SD)*	*N*	*Mean (SD)*	*NS*
Mean age (years)	189	28.7 (5.6)	98	31.10 (4.70)	NS
Mean pre-pregnancy BMI (kg/m^2^)	112	27.1 (6.6)	99	31.10 (4.18)	NS
*Categorical variables:*	*N (%)*		*N (%)*		
Mode of delivery					NS
Vaginal delivery	114 (65)		67 (67.68)		
Caesarian section	63 (35)		32 (32.32)		
Chorioamnionitis Diagnosis					N/A
Yes	10 (6)		0 (N/A)		
No	169 (94)		81 (82)		
Smoking status					*p* < 0.001
Current smokers	28 (15)		1 (1.01)		
Former/never smokers	148 (85)		97 (97.98)		
	**United States Infant Population**		**Nigerian Infant Population**		
*Continuous variables:*	*N*	*Mean (SD)*	*N*	*Mean (SD)*	
Gestational age at delivery (weeks of gestation)	189	38.04 (3.1)	99	38.40 (2.35)	NS
*Infant birth anthropometrics:*					NS
Birth weight (g)	189	3109.8 (783.4)	99	3086.21 (479.13)	
Birth length (cm)	189	48.43 (4.7)	99	49.25 (3.79)	
Birth head circumference (cm)	189	33.50 (2.8)	97	34.37 (2.36)	
*Categorical variables:*	*N (%)*		*N (%)*		
Premature	34 (18)		15 (15.2)		NS
Gender					NS
Male (%)	96 (51)		50 (50.51)		
Female (%)	93 (49)		49 (49.49)		

BMI—body mass index; NS—not significant; N/A: not applicable.

**Table 2:** Maternal and cord plasma tocopherol levels *.

	Median Vitamin E Tocopherol Levels (mcg/L) (Range)		
	United States Cohort	Nigerian Cohort	*p*-Value
Maternal α-tocopherol	12,375.85 (175.23–34,687.75)	8333.08 (1576.59–16,248.40)	<0.001
Maternal γ-tocopherol	1340.73 (224.59–4385.95)	357.51 (66.36–1775.31)	<0.001
Maternal δ-tocopherol	261.71 (24.70–1324.71)	368.94 (43.06–1886.47)	<0.001
Maternal α:γ-tocopherol ratio	8.54 (0.20–44.39)	26.19 (4.37–65.35)	<0.001
Cord α-tocopherol	1861.17 (80.56–6958.85)	1923.26 (728.07–12,885.87)	0.383
Cord γ-tocopherol	203.13 (42.53–1953.23)	113.87 (0.00–823.00)	<0.0001
Cord δ-tocopherol	74.51 (16.32–716.52)	88.86 (0.00–958.64)	0.379
Cord α:γ-tocopherol ratio	8.91 (0.49–44.93)	18.77 (1.48–91.62)	<0.001

* Statistical method used: Mann–Whitney U test.

**Table 3:** United States (US) maternal and cord plasma tocopherol levels by mode of delivery *.

	Median Tocopherol Levels (mcg/L) (Range)			Median Tocopherol Levels (mcg/L) (Range)		
	US Vaginal Delivery	US Caesarean Section	*p*-Value	Nigerian Vaginal Delivery	Nigerian Caesarean Section	*p*-Value
Maternal α-tocopherol	12,332.01 (175.23–34,687.75)	12,487.40 (2269.46–20,190.35)	0.870	8555.84 (1576.59–13,382.37)	7766.52 (1801.04–16,248.40)	0.092
Maternal γ-tocopherol	1269.06 (224.59–3604.48)	1413.52 (151.01–4385.95)	0.028	314.57 (66.36–1574.22)	445.36 (141.18–1775.31)	0.003
Maternal δ-tocopherol	260.81 (24.70–1324.71)	260.56 (34.80–780.33)	0.430	361.82 (43.06–1071.62)	387.57 (93.16–1886.47)	0.356
Maternal α:γ-tocopherol ratio	9.23 (0.20–44.39)	7.81 (2.20–34.62)	0.013	27.38 (5.26–65.35)	15.03 (4.37–61.27)	<0.001
Cord α-tocopherol	1804.32 (80.56–6958.85)	1976.08 (899.73–3991.82)	0.270	1848.86 (867.92–12,885.87)	2185.74 (728.07–7387.90)	0.272
Cord γ-tocopherol	199.07 (42.53–1953.23)	215.12 (71.63–575.06)	0.350	82.54 (0.00–823.00)	225.78 (37.00–553.75)	<0.001
Cord δ-tocopherol	77.98 (0.00–573.64)	60.31 (0.00–298.27)	0.170	73.62 (0.00–517.19)	114.76 (24.45–958.64)	0.0002
Cord α:γ-tocopherol ratio	8.91 (0.49–44.93)	8.97 (3.97–25.33)	0.941	27.26 (1.48–91.62)	10.60 (2.42–64.63)	<0.001

* Statistical method used: Mann–Whitney U test.

**Table 4:** United States maternal and cord plasma tocopherol levels by diagnosis of chorioamnionitis *.

	Median Tocopherol Levels (mcg/L) (Range)		
	Chorioamnionitis Diagnosis		*p*-Value
	Yes	No	
Maternal α-tocopherol	9629.57 (4185.93–16,389.15)	12,498.16 (175.23–34,687.75)	0.058
Maternal γ-tocopherol	1054.20 (311.58–2248.52)	1347.84 (224.59–4385.95)	0.051
Maternal δ-tocopherol	185.93 (27.87–403.94)	262.76 (24.70–1324.71)	0.186
Cord α-tocopherol	1972.37 (1219.44–2911.61)	1861.17 (80.56–6958.85)	0.773
Cord γ-tocopherol	178.57 (63.75–231.58)	203.78 (42.53–1953.23)	0.108
Cord δ-tocopherol	93.51 (45.71–157.39)	74.34 (16.32–716.52)	0.582

* Statistical method used: Mann–Whitney U test.

**Table 5:** United States infant population plasma tocopherol levels by infant growth parameter z-scores less than two standard deviations below the mean vs. two standard deviations or more below the mean.

Median Tocopherol Levels (mcg/L) (Range) as Compared to Infants by Z-Score ≤−2 vs. >−2 *												
	Weight-for-Length Z-Score		*p*-Value	Weight-for-Age Z-Score		*p*-Value	Length-for-Age Z-Score		*p*-Value	Head-Circumference-for-Age Z-Score		*p*-Value
	≤−2	>−2		≤−2	>−2		≤−2	>−2		≤−2	>−2	
US cord α-tocopherol	2224.02 (1273.45–4615.08)	1793.69 (80.56–4514.84)	0.244	2223.21 (899.73–6958.85)	1793.69 (80.56–4514.84)	0.009	2362.06 (1380.02–6958.85)	1746.48 (80.56–4514.84)	0.006	1833.72 (1219.44–6958.85)	1888.18 (80.56–4615.08)	0.50
US cord γ-tocopherol	251.29 (83.20–524.85)	198.13 (42.53–652.87)	0.133	328.19 (77.92–1953.23)	195.91 (42.53–652.87)	<0.001	261.66 (73.94–1953.23)	197.08 (42.53–652.87)	0.064	220.71 (73.94–1953.23)	199.45 (42.53–652.87)	0.18
US cord δ-tocopherol	80.89 (29.86–454.64)	74.34 (16.32–709.50)	0.169	74.61 (31.23–716.52)	73.16 (16.32–709.50)	0.264	55.19 (31.23–716.52)	75.54 (16.32–709.50)	0.338	79.11 (30.91–716.52)	72.26 (16.32–709.50)	0.35
Nigeria cord α-tocopherol	1737.66 (907.16–12,885.87)	1945.30 (728.07–9359.80)	0.265	2212.43 (1104.84–5871.71)	1901.35 (728.07–12,885.87)	0.835	1785.19 (867.92–2393.97)	1945.30 (728.07–12,885.87)	0.124	2149.88 (728.07–6905.18)	1864.00 (867.92–9359.80)	0.322
Nigeria cord γ-tocopherol	243.77 (13.67–823.00)	96.95 (0.00–699.43)	0.177	59.71 (13.67–553.75)	122.88 (0.00–823.00)	0.098	71.54 (0.00–271.29)	133.51 (26.02–823.00)	0.003	178.19 (57.09–553.75)	109.19 (0.00–823.00)	0.296
Nigeria cord δ-tocopherol	80.92 (15.61–958.64)	88.84 (0.00–517.19)	0.923	56.80 (15.61–958.64)	89.85 (0.00–517.19)	0.342	75.56 (0.00–144.20)	88.88 (13.98–958.64)	0.100	138.12 (70.12–958.64)	84.77 (0.00–517.19)	0.034

* Statistical method used: Mann–Whitney U test.
